# The impact of magnetized cold plasma and its various properties in sensing applications

**DOI:** 10.1038/s41598-022-07461-4

**Published:** 2022-03-08

**Authors:** Zaky A. Zaky, Abinash Panda, Puspa D. Pukhrambam, Arafa H. Aly

**Affiliations:** 1 TH-PPM Group, Physics Department, Faculty of Science, Beni-Suef University, Beni-Suef, 62521 Egypt; 2 Department of Electronics and Communication Engineering, National Institute of Technology, Silchar, Assam 788010 India

**Keywords:** Biophotonics, Magneto-optics, Nanophotonics and plasmonics, Terahertz optics

## Abstract

These analyses present a novel magnetized cold plasma-based 1D photonic crystal structure for detecting the refractive index of various bio-analytes. The proposed structure is designed with two photonic crystals composed of an alternating layer of right-hand polarization and left-hand polarization of the magnetized cold plasma material with a central defect layer. Transmittance characteristics of the structure are studied by employing the well-known transfer matrix method. Various geometrical parameters such as electron density, external magnetic field, thickness of odd and even layers of the multilayers, thickness of the sample layer, and incident angle are judiciously optimized to attain the best sensitivity, figure of merit, quality factor, signal-to-noise ratio, detection range and limit of detection. Finally, a maximum sensitivity of 25 GHz/RIU is accomplished with the optimized value of structure parameters, which can be considered as a noteworthy sensing performance.

## Introduction

In late 1987, Yablanovitch and John^1,2^ presented the first concept of photonic crystal (PhC) structure. They described PhC as a periodic arrangement of dielectric materials, where the periodicity may be in one-dimension, two-dimensions, and three-dimensions^3–6^. Since then, intensive research on PhC has been going on^7–17^. In the last decade, PhC structures have emerged as suitable candidates to realize a variety of modern-day significant applications in optical engineering like biosensors, filters, etc.^18–24^. The foremost beauty of PhC is its ability to control the flow of electromagnetic waves inside it, which leads to the prevention of certain wavelength range passing through the structure. This range of wavelength is known as the photonic bandgap (PBG)^25,26^. Introducing a defect layer into the periodic PhC can greatly influence the PBG forming a narrow defect mode^27^. Studying the position and intensity of the defect mode can open up a new horizon of research opportunities in optical filters and biosensors.

Recently, refractive index-based biosensors using PhC have gathered enormous research importance owing to their enhanced sensitivity, broad dynamic range, and low-cost fabrication techniques^15,28,29^. Refractive index of bio-analytes is studied as a noteworthy biophysical parameter for cutting-edge biosensing applications. The defect mode wavelength and intensity are highly sensitive to a small change in the refractive index of biomolecules^30^. Therefore, by precisely measuring the refractive index, it is possible to easily detect different concentrations of biomolecules in real-time and quickly.

In the recent decade, researchers have explored many peculiar properties of PhC by designing the structure with metal, semiconductor, superconductor, metamaterial, gyroidal, etc.^31–35^. Apart from this, a new kind of material is known as magnetized cold plasma (MCP). MCP has recently gained a lot of attention owing to its unique optical, electrical and mechanical properties^36^. MCP is an exceptional material, which shows externally controlled electric permittivity, and its physical properties can be suitably handled by varying external parameters like an external magnetic field^37^. Under the influence of an external magnetic field, MCP material shows an interesting property known as gyro-effective frequency. The right-hand polarization (RHP) and left-hand polarization (LHP) of MCP indicate positive and negative values of gyro-effective frequency, respectively^38,39^. RHP and LHP exist in the MCP under the presence of positive and negative values of the external applied magnetic field, respectively. Various parameters like electron density, magnetic field, collision frequency, etc. can be effectively managed to regulate the refractive index of MCP material. Owing to the aforementioned property of MCP, it can be considered as a good alternative to conventional metal and dielectric materials vis-à-vis the study and fabrication of PhC^40,41^. The first concept of MCP material was introduced by Hojo et al.^42^, where the authors demonstrated the PBG characteristics in a magnetized plasma-based photonic crystal (MCPPhC). Further, they investigated the effect of plasma density on the transmission spectrum and found the wavelength corresponding to the total PBG. Besides, an external magnetic field is applied across the MCPPhC to achieve PBG, which leads to the realization of a novel type of PhC known as MCPPhC. Owing to its captivating optical properties compared to the traditional PhC, MCPPhC has been the center of PhC research^43^.

Awasthi et al. demonstrated a one-dimensional MCPPhC (1D-MCPPhC) in the microwave region. The authors investigated the reflectance characteristics concerning variation in the external magnetic field to realize multichannel tunable PBG^36^. Chittaranjan et al. envisaged a multichannel microwave reflector by studying the transmittance spectrum of octonacci 1D-MCPPhC^44^. They revealed that a higher number of PBGs can be obtained with the optimized value of electron density, thickness ratio and external magnetic field^44^. A. H. Aly et al. reported a defective 1D-PhC, where the authors considered MCP as the defect layer^41^. In addition, the impact of the external magnetic field on the defect mode properties is explained. Aghajamali et al. studied the transmittance characteristics of a hybrid PhC composed of alternate layers of superconductor and MCP material^45^. Aly et al. demonstrated a clear view of the magnetic field on the transmission spectrum of a defected 1D-PhC in ultraviolet wavelength^46^. A. Kumar et al. realized a tunable broadband reflector and the narrowband filter using a 1D-PhC designed with MCP and dielectric material. They employed the transfer matrix method (TMM) and dispersion relation to investigate the transmittance spectra for both RHP and LHP conditions^47^. Nevertheless, a healthy number of researches have been carried out by selecting MCP material for multichannel filtering application in the microwave region^48,49^, but sensing application using 1D-MCPPhC is still not properly explored.

The present work has a certain uniqueness. It presents a detailed analysis of sensing performances of periodic MCP layers. Besides, we aim to know if the periodicity of the magnetic property of layers (gyro-effective frequency) will add an additional property to the sensor or not. Moreover, complete optimization of various structural parameters such as electron density, external magnetic field, thickness of odd and even layers of the MCPPhC, thickness of the sample layer, and incident angle are studied in this work, which has not been investigated so far in any of the previously published articles. Both RHP and LHP characteristics of the magnetized plasma material are studied, which further enhances the novelty. Moreover, high sensing performance such as high sensitivity, figure of merit, quality factor, signal-to-noise ratio, detection range, very small limit of detection and resolution makes the proposed structure a suitable candidate for biosensing applications.

## Proposed structure and theoretical model

**Figure 1:**
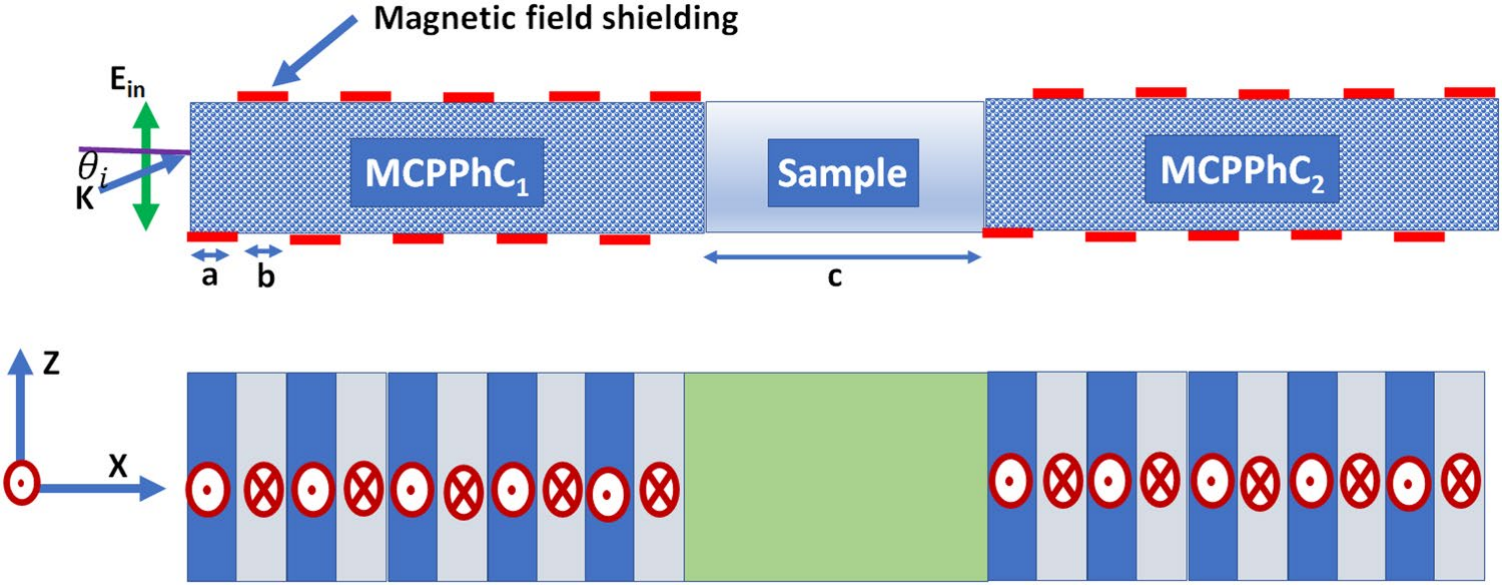
The schematic representation of the defected 1D-MCPPhC sensor.

The proposed 1D-MCPPhC sensor is delineated in Fig. 1. It composes of PhC_1_ and PhC_2_ with a defect layer sandwiched between them. In both MCPPhC_1_ and MCPPhC_2_, the first layer is MCP under the magnetic field in the negative y-direction (MCP^-^), whereas the second layer is designed with the MCP layer under the magnetic field in the positive y-direction (MCP^+^). The thickness of the first and second layers are denoted as $a$ and $b$, whereas the defect layer thickness is symbolized as $c$. The proposed sensor is realized with a configuration (MCP^-^/MCP^+^)^N^/defect/(MCP^-^/MCP^+^)^N^. Different liquid samples with small variations in refractive index from 1.0 to 1.1 are infiltrated within the central defect layer. We assume a normal and oblique light penetrates the proposed structure.

The permittivity of the MCP layer as a function of external magnetic field and frequency in gigahertz range can be calculated as^36,40,45,50,51^:

1 $${\upvarepsilon }_{R}^{L}\left(\upomega \right)=1-\left[\frac{{\upomega }_{p}^{2}}{{\upomega }^{2}\left[1 - \frac{i\gamma }{\upomega } \mp \frac{{\upomega }_{gy}}{\upomega }\right]}\right],$$

where $\upomega$, ${\upomega }_{p}$, ${\upomega }_{gy}$, and $\gamma$ are angular frequency, plasma frequency, gyrofrequency, and effective collision frequency, respectively. plasma frequency and gyrofrequency can be calculated as:

2 $${\upomega }_{p}=\sqrt{\left(\frac{{n}_{e} {e}^{2}}{{m}_{e} {\upvarepsilon }_{0}}\right)},$$

3 $${\upomega }_{gy}=\frac{e B}{{m}_{e} },$$

where ${n}_{e}, {m}_{e}, {\upvarepsilon }_{0}$, e and $B$ are electron density, electron mass, vacuum permittivity, electronic charge, and external magnetic field, respectively. The minus sign of ($\mp )$ in Eq. (1) denotes the right-hand polarization (RHP) and means the magnetic field is in the positive y-direction. This is the condition of the second layer of the PhC in Fig. 1. For the first layer of the PhC, ${\upomega }_{gy}$ takes a positive sign and refers to LHP. In our study, we assumed that the temperature keeps constant. So, the effective refractive indices will not be affected by temperature.

To simplify the manuscript, we focus only on the TE mode wave, where the electric field vector is perpendicular to the plane of incidence. The incoming electric field (${E}_{\mathrm{i}}$) and magnetic field (${H}_{\mathrm{i}}$) components are mathematically related to transmitted field intensities (${E}_{\mathrm{t }}\mathrm{and} {H}_{t}$) as stated below:

4 $$\left[\begin{array}{c}{E}_{i}\\ {H}_{i}\end{array}\right]={M}_{1},{M}_{2},{M}_{3},\dots ..{M}_{N}\left[\begin{array}{c}{E}_{t}\\ {H}_{t}\end{array}\right],$$

where M denotes the characteristics matrix, and N signifies the total number of layers. To study the transmission spectrum of the suggested structure, we have employed the TMM. The characteristics matrix or the transfer matrix representation for a single layer (P) can be expressed as^52^:

5 $${M}_{P}=\left[\begin{array}{cc}\mathrm{cos}({k}_{xP}{d}_{s})& \frac{i}{{{ \emptyset }}_{P}}sin({k}_{xP}{d}_{P}) \\ i{{ \emptyset }}_{P}({k}_{xP}{d}_{P})& cos({k}_{xP}{d}_{P})\end{array}\right], P=A,B,D,$$

where, ${k}_{xP}={k}_{0}\sqrt{{\varepsilon }_{P}-{sin}^{2}({\theta }_{i})}$, signifies the wave number of *P*th layer, ${k}_{0}=\frac{\omega }{c}$ represents the wave vector for vacuum, ${d}_{P}$ denotes the thickness of *P*th layer and ${{ \emptyset }}_{s}$ is defined as $\frac{{k}_{xP}}{{k}_{0}}$.

The overall transfer matrix (M) can be evaluated by multiplying the transfer matrix of individual layers, which is stated as:

6 $$M=\left(\begin{array}{cc}{M}_{11}& {M}_{12}\\ {M}_{21}& {M}_{22}\end{array}\right)={\left({M}_{a}{M}_{b}\right)}^{N}{M}_{c}{\left({M}_{a}{M}_{b}\right)}^{N}.$$

In Eq. (6), ${M}_{a}$, ${M}_{b}$ and ${M}_{c}$ represent the characteristic matrices of the layers a, b and c, respectively. The elements of the matrix obtained in the above equations can be used to compute the transmission coefficient, which can be specified as^53^:

7 $$t=\frac{2{ \emptyset }_{0}}{\left({M}_{11}+{M}_{12}{ \emptyset }_{s}\right){ \emptyset }_{0}+\left({M}_{21}+{M}_{22}{ \emptyset }_{s}\right)},$$

where ${ \emptyset }_{p}={n}_{p}\mathrm{cos}({\theta }_{p}).$ Transmittance (T) of the complete structure is expressed as:

8 $$T=\frac{{ \emptyset }_{1}}{{ \emptyset }_{0}}\left|{t}^{2}\right|.$$

The most significant parameters to appraise the performance of any sensor are sensitivity (S), figure of merit (FoM), Quality factor (Q), signal-to-noise ratio (SNR), detection range (DR), limit of detection (LoD), and RS (sensor resolution). These sensing parameters can be mathematically expressed as^54^:

9 $$S=\frac{\Delta {f}_{R}}{\Delta {n}_{s}},$$

10 $$FoM=\frac{S}{FWHM},$$

11 $$\begin{array}{l}\mathrm{Q}=\frac{{f}_{\mathrm{R}}}{\mathrm{FWHM}}\end{array},$$

12 $$\mathrm{SNR}=\frac{\Delta {f}_{\mathrm{R}}}{\mathrm{FWHM}},$$

13 $$\mathrm{RS}=\frac{2\left(\mathrm{FWHM}\right)}{{3\left(\mathrm{SNR}\right)}^\frac{1}{4}},$$

14 $$\begin{array}{l}\mathrm{L}o\mathrm{D}=\frac{{f}_{\mathrm{R}}}{20\mathrm{ S Q}},\end{array}$$

15 $$\begin{array}{c}\mathrm{DR}=\frac{{f}_{\mathrm{R}}}{\sqrt{\mathrm{FWHM}}}.\end{array}$$

The sensitivity is defined as the ratio of the frequency shift of the transmittance peak to the change in the refractive index of the sample. FoM guarantees that the sensitivity is increased while the FWHM is reduced. The ratio of the resonant peak frequency to the FWHM is called Q. The SNR is the ratio of the peak's frequency shift to the FWHM. The minimal frequency peak variation that can be noticed is known as RS. The minimal change in the analyte refractive index that may be measured is called LoD.

### Ethics declarations

This article does not contain any studies involving animals or human participants performed by any of the authors.

## Results and discussions

In this section, the detailed performance analysis of the proposed sensor will be demonstrated for different thicknesses of the MCP layer, different thicknesses of defect layer, different angles of incidence and different values of the external magnetic field across the MCP layer. Initially, the thickness of different layers is selected as $a=15 \mathrm{mm}$, $b=15 \mathrm{mm}$, and $c=50 \mathrm{mm}$. The value of ${n}_{e}$ is $8\times {10}^{17}{\mathrm{m}}^{-3}$, whereas the value of $\upgamma$ is taken as $4\pi \times {10}^{4}$^40^. The value of $\upgamma$ is considered as a constant because the collision frequency ($\upgamma )$ has a negligible impact on the resonant peaks^55,56^. The number of periods of each PhC is set as N = 5, and the substrate is considered as air medium, which has a refractive index of 1.0.

**Figure 2:**
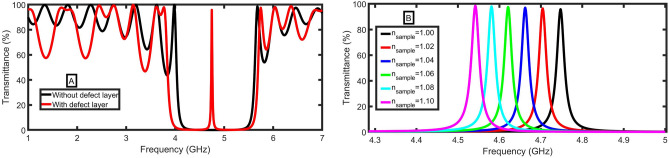
The transmittance of the MCP-PhC structure (**A**) without and with the sample layer (n_sample_ = 1.0) (**B**) for different values of n_sample_ at B = 1 T.

The importance of the defect layer in the proposed structure will be investigated, which is shown in Fig. 2A. With the presence of the defect layer, the width of PBG increases, and the resonant mode is formed within the PBG compared to the case of the absence of the defect layer. The creation of a defect layer forms the basis for sensing application. As clear in Fig. 2B, the shift in resonant mode wavelength can be computed concerning different sensing analyte refractive indices. The defect mode is red-shifted (is shifted to lower frequencies) as the refractive index of the analyte sample increases. More specifically, the defect mode frequency decreases from 4.75 to 4.54 GHz, with an increase in refractive index from 1.0 to 1.1. This significant shift ensures a good sensing characteristic.

### Effect of electron density

**Figure 3:**
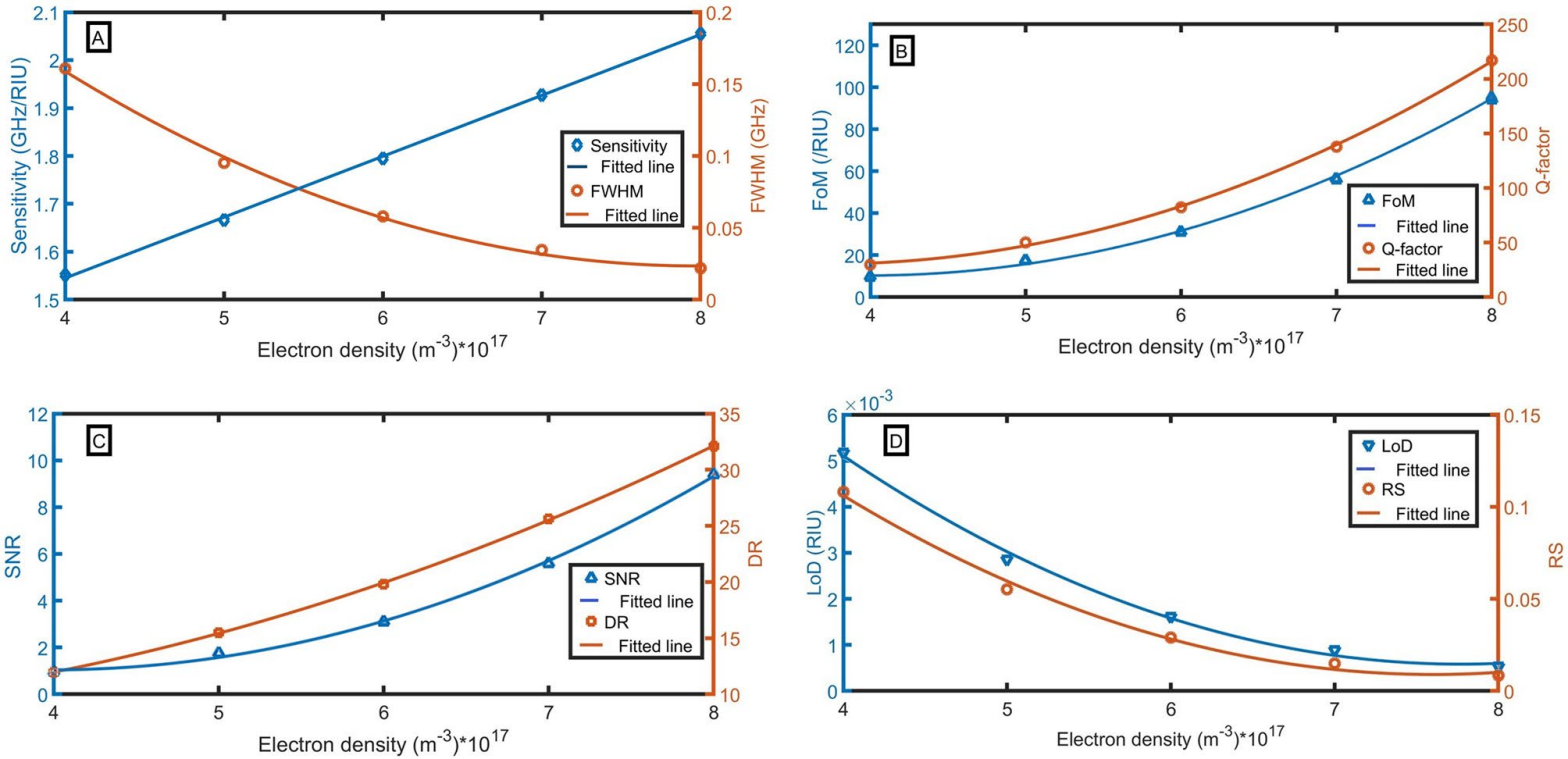
Evaluation of the sensor as a function of electron density with changing the sample refractive index from 1.0 to 1.1 at B = 1 T, (**A**) sensitivity and FWHM (for n = 1.1), (**B**) FoM and Q-factor, (**C**) SNR and DR, and (**D**) LoD and RS.

The sensitivity, FoM, Q-factor, SNR, RD, LoD, and RS are thoroughly analyzed for different values of electron density from $4\times {10}^{17}{ \mathrm{m}}^{-3}$ to $8\times {10}^{17}{ \mathrm{m}}^{-3}$. We stopped the study at $8\times {10}^{17}{ \mathrm{m}}^{-3}$ according to this reference^45^. In Fig. 3A, the sensitivity increases linearly with the increase of electron density according to the following fitted equation:

16 $${\text{S }} = { }1.269\times {10}^{-18}n_{{\text{e}}} + { }1.0374,{ }\left( {{\text{R}}^{{2}} = 0.9994} \right).$$

A maximum sensitivity of 2.055 GHz/RIU is obtained at ${n}_{e}=8\times {10}^{17}{ \mathrm{m}}^{-3}$. Additionally, as FWHM is lowest at this value of ${n}_{e}$, therefore optimum FoM, Q-factor, SNR, RD, LoD, and RS are attained at ${n}_{e}=8\times {10}^{17}{ \mathrm{m}}^{-3}$. The following equations describe the quadratic relation between the electron density and FWHM, FoM, Q-factor, SNR, RD, LoD, and RS:

17 $${\text{FWHM }} = { }8.5608\times {10}^{-37}{ }n_{e}^{2} - { }1.3658\times {10}^{-18}n_{{\text{e}}} + 5.6821,{ }\left( {{\text{R}}^{2} { } = { }0.9974} \right),$$

18 $${\text{FoM }} = { }5.13126\times {10}^{-34}n_{e}^{2} - { }4.08705\times {10}^{-16}n_{{\text{e}}} + { }91.8413,{ }\left( {{\text{R}}^{{2}} { } = { }0.9987} \right),$$

19 $${\text{Q}} = 1.00743\times {10}^{-33}n_{e}^{2} - 7.46607\times {10}^{-16}n_{e} + 168.62,\,\,({\text{R}}^{2} = 0.9993),$$

20 $${\text{SNR}} = 5.13126\times {10}^{-35}n_{e}^{2} - 4.08705\times {10}^{-17}n_{{\text{e}}} + 9.1841,\,\,({\text{R}}^{2} = 0.9987),$$

21 $${\text{DR}} = 5.28444\times {10}^{-35}n_{e}^{2} - 1.29896\times {10}^{-17}n_{e} + 8.70648,\,\,({\text{R}}^{2} = 0.9999),$$

22 $${\text{LoD}} = 3.18132\times {10}^{-38}n_{e}^{2} - 4.94391\times {10}^{-20}n_{e} + 0.01979,\,\,({\text{R}}^{2} = 0.9961),$$

23 $${\text{RS}} = 7.47651\times {10}^{-37}n_{e}^{2} - 1.13704\times {10}^{-18}n_{e} + 0.441248,\,\,({\text{R}}^{2} = 0.9941).$$

From Fig. 3B–D, we perceived a maximum FoM, Q-factor, SNR, DR of 93.98 /RIU, 217.11, 9.40, 32.11 respectively. Also, a very small LoD and RS of $5.3\times {10}^{-4}$ RIU and 8.3 $\times {10}^{-3}$ is attained respectively. Therefore, the most suitable value of ${n}_{e}$ is selected as $8\times {10}^{17}{ \mathrm{m}}^{-3}$.

### Effect of external magnetic field (B)

**Figure 4:**
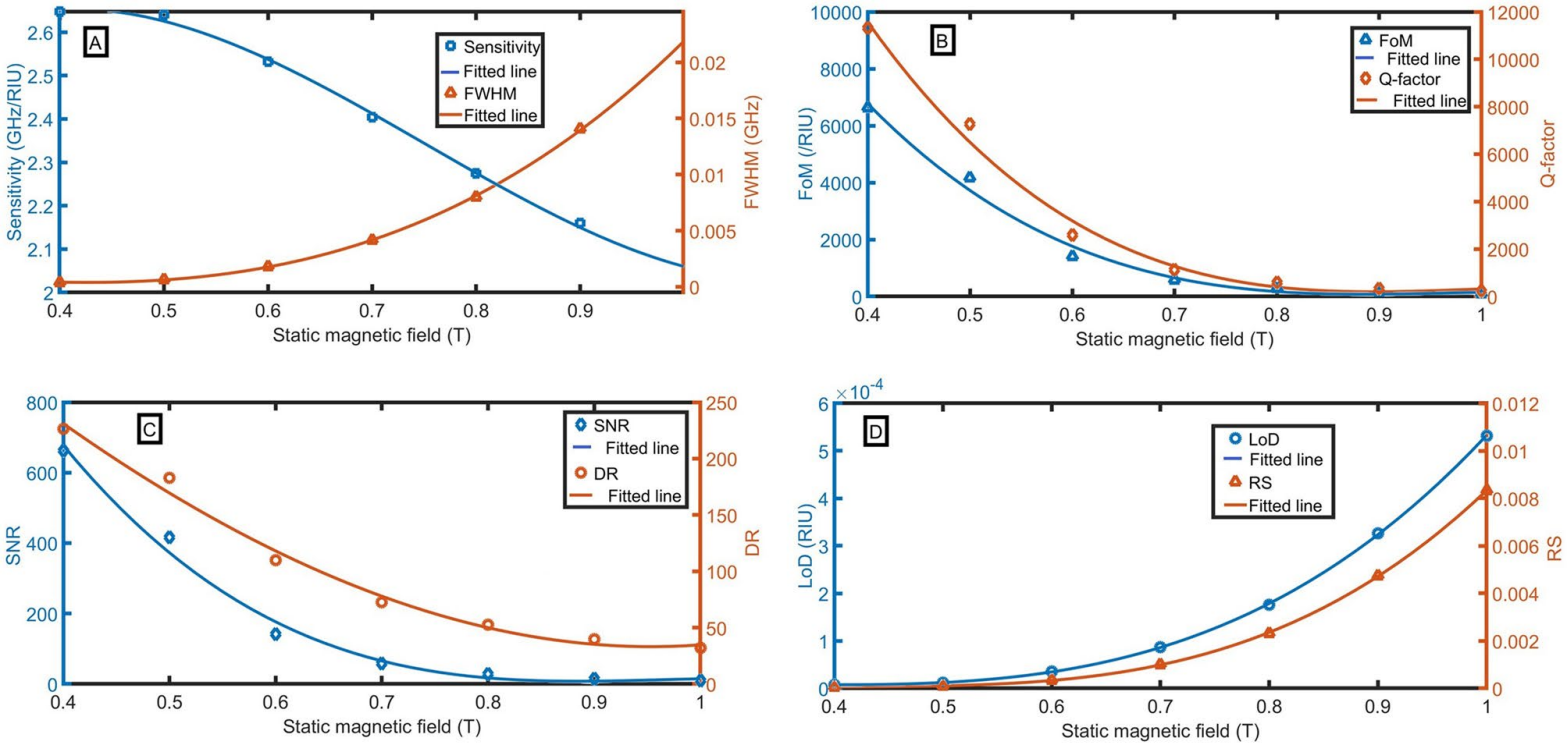
Evaluation of the sensor as a function of the external magnetic field with changing the sample refractive index from 1.0 to 1.1 at n_e_ = 8*${10}^{17}$ ${m}^{-3}$, (**A**) sensitivity and FWHM (for n = 1.1), (**B**) FoM and Q-factor, (**C**) SNR and DR, and (**D**) LoD and RS.

By keeping ${n}_{e}=8\times {10}^{17}{ \mathrm{m}}^{-3}$ constant, we applied different magnetic fields from 0.4 to 1 T across the cold plasma layer and computed various sensing performance parameters for different sample refractive indices from 1.0 to 1.1 in the defect layer, which are delineated in Fig. 4A–D. The performance of the sensor decreases with the magnitude of the magnetic field increase. The following equations describe the cubic relation between the magnetic field and S, FWHM, FoM, Q-factor, SNR, RD, LoD and RS:

24 $${\text{S}} = 4.0278B^{3} - 9.0857B^{2} + 5.4483B + 1.6693,\,\,({\text{R}}^{2} = 0.9985),$$

25 $${\text{FWHM}} = 0.0501B^{3} - 0.0274B^{2} - 0.0039B + 0.0032,\,\,\,({\text{R}}^{2} = 0.9999),$$

26 $${\text{FoM}} = - 38662B^{3} + 112390B^{2} - 108078B + 34500,\,\,({\text{R}}^{2} = 0.9905),$$

27 $${\text{Q}} = - 60276B^{3} + 178527B^{2} - 174676B + 56742,\,\,({\text{R}}^{2} = 0.9902),$$

28 $${\text{SNR}} = - 3866.2B^{3} + 11239B^{2} - 10808B + 3450,\,\,({\text{R}}^{2} = 0.9905),$$

29 $${\text{DR}} = - 167.49B^{3} + 259.81B^{2} - 952.22B + 559.64,\,\,({\text{R}}^{2} = 0.9902),$$

30 $${\text{LoD}} = 0.0019B^{3} - 0.002B^{2} + 0.0007B - 7 \times 10^{ - 5} ,\,\,\,(R^{2} = 0.9999),$$

31 $${\text{RS}} = 0.046B^{3} - 0.0611B^{2} + 0.0275B - 0.0041,\,\,({\text{R}}^{2} = 0.9999).$$

Even though the highest performance is observed at B = 0.4 T, the resonant peak has very low intensity. At B = 0.4 T, the sensitivity and transmittance are 2.647 GHz/RIU and 28%. At B = 0.5 T, the sensitivity and transmittance are 2.640 GHz/RIU and 71%. B = 0.4 T slightly increases the sensitivity, but B = 0.5 T strongly increases the transmittance of resonant peaks. Therefore, the optimized value of the external magnetic field is B = 0.5 T. The sensitivity, FoM, Q-factor, SNR, DR, LoD, and RS of 2.64 GHz/RIU, $4168$ /RIU, $7265$, 417, 183, $1\times {10}^{-5}$ RIU, $9\times {10}^{-5}$ are obtained respectively at the external magnetic field of 0.4 T across the plasma layer.

### Effect of thickness of sample layer (c)

**Figure 5:**
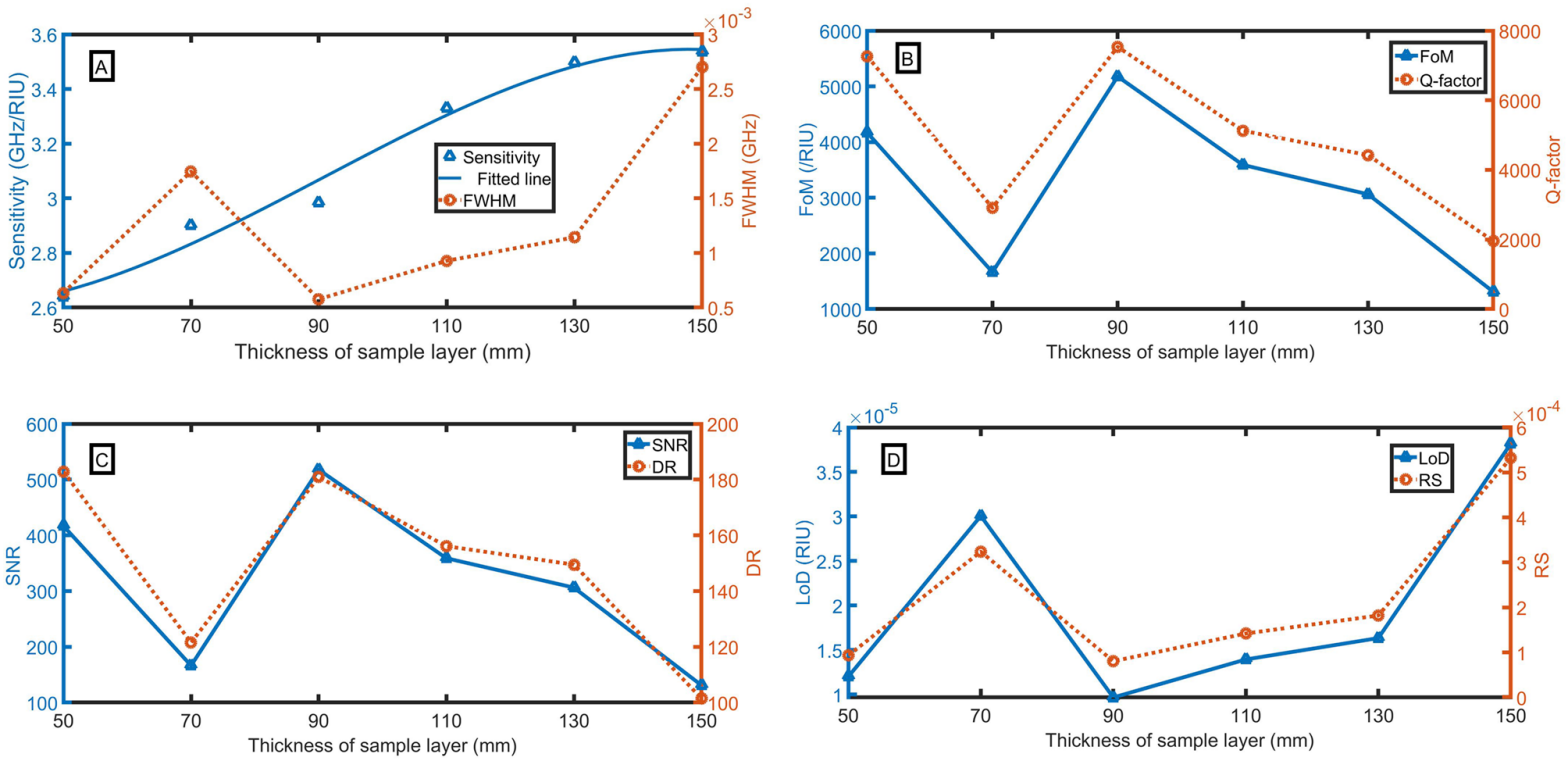
Evaluation of the sensor performance as a function of the thickness of the sample layer with changing the sample refractive index from 1.0 to 1.1 at n_e_ = 8 × 10^17^ m^-3^ and B = 0.4 T, (**A**) sensitivity and FWHM (for n = 1.1), (**B**) FoM and Q-factor, (**C**) SNR and DR, and (**D**) LoD and RS.

The thickness of the sample layer (c) plays a significant role in optimizing the sensor performance. By changing the value of (c) over a wide range from 50 to 150 mm, various performances are computed as clear in Fig. 5A–D. It is seen that the performance varies nonlinearly with an increase in the sample layer thickness. In Fig. 5A, the sensitivity increases quadrically with the increase of sample layer thickness according to the following fitted equation:

32 $${\text{S}} = 10^{ - 6} c^{3} + 0.0003c^{2} - 0.0183c + 2.8863,\,\,({\text{R}}^{2} = 0.9801).$$

Even though the sensitivity at thickness of 150 mm (3.536 GHz/RIU) is slightly higher than at the thickness of 130 mm (3.498 GHz/RIU), the FWHM at the thickness of 130 mm (0.0011 RIU^−1^) is strongly lower than at the thickness of 150 mm (0.0027 RIU). As the enhancement in the FWHM is higher than the enhancement in the sensitivity. So, the optimum value of (c) is considered 130 mm. Satisfactory sensing performances such as sensitivity, FoM, Q-factor, SNR, DR, LoD, and RS of 3.50 GHz/RIU, $3062$ /RIU, $4422$, 306, 150, $2\times {10}^{-5}$ RIU, $2\times {10}^{-4}$ are attained respectively.

### Effect of thickness of first MCP layer of the PhC (a)

**Figure 6:**
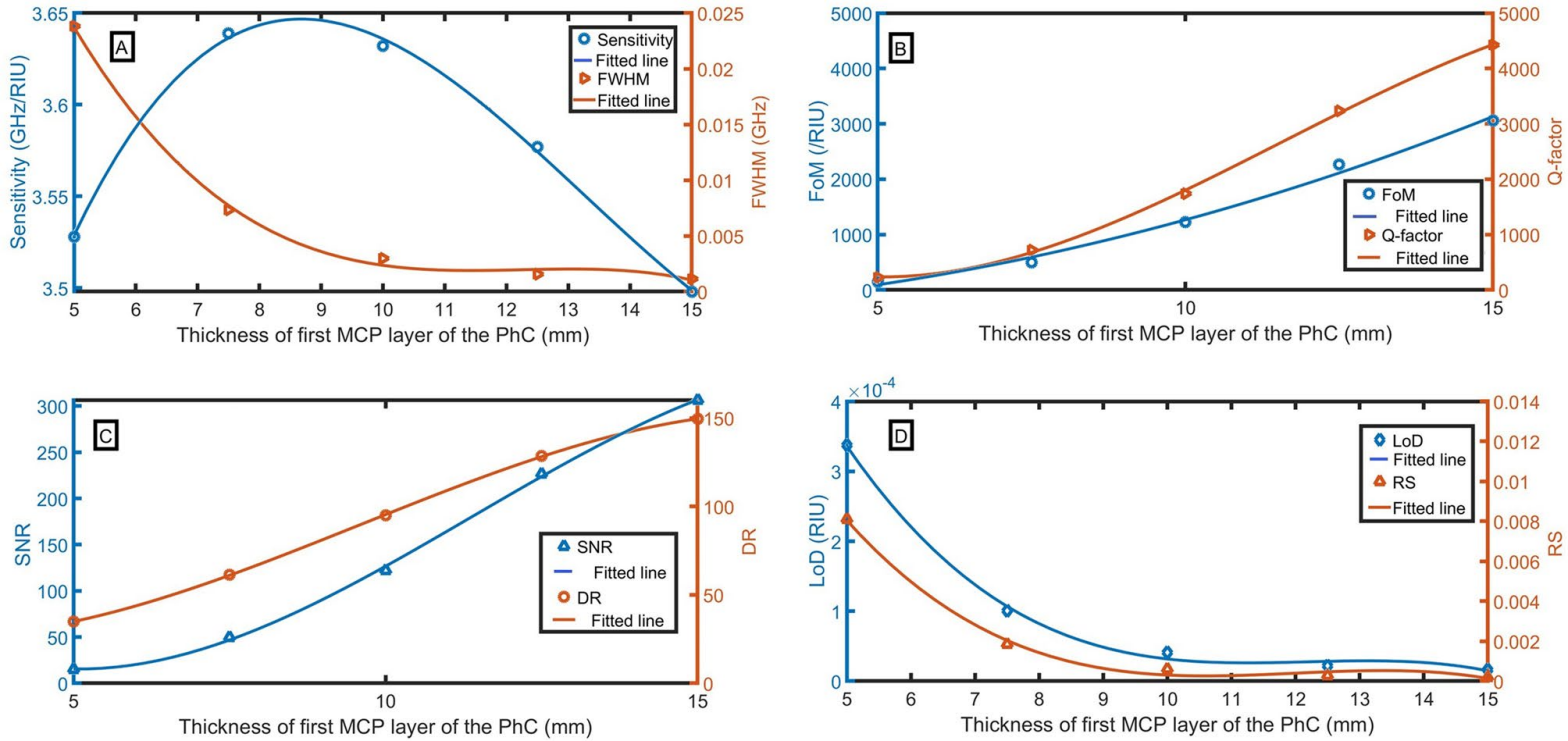
Evaluation of the sensor as a function of the thickness of the first MCP layer of the PhC with changing the sample refractive index from 1.0 to 1.1, (**A**) sensitivity and FWHM (for n = 1.1), (**B**) FoM and Q-factor, (**C**) SNR and DR, and (**D**) LoD and RS.

Further, we systematically study the effect of thickness of odd layers of MCPPhC (a), which is depicted in Fig. 6A–D. It is observed that the performance is greatly influenced by the thickness of the odd layers of the proposed MCPPhC. Different performance parameters are studied by changing the thickness(a) from 5 to 15 mm and found that the performance of the proposed sensor is improved with the increase of the thickness (a). Even though the sensitivity at the thickness of 7.5 mm (3.639 GHz/RIU) is slightly higher than at the thickness of 15 mm (3.498 GHz/RIU), the FWHM at the thickness of 15 mm (0.0011 RIU^−1^) is strongly lower than at the thickness of 7.5 mm (0.0073 RIU^−1^). As the enhancement in the FWHM is higher than the enhancement in the sensitivity. So, the optimum value is considered 15 mm. By increasing the thickness higher than 15 mm, the resonant peak goes out from the PBG. The following equations describe the relationship between the thickness of the first MCP layer of the PhC and S, FWHM, FoM, Q-factor, SNR, RD, LoD and RS:

33 $${\text{S}} = 5.01333 \times 10^{ - 4} a^{3} - 1.99314\times 10^{ - 2}a^{2} + 2.32695\times 10^{ - 1}a + 2.8008,\,\,({\text{R}}^{2} = 0.998),$$

34 $${\text{FWHM}} = - 5.96728 \times 10^{ - 5} a^{3} + 2.19113\times 10^{ - 3}a^{2} - 2.66967\times 10^{ - 2}a + 1.09882\times 10^{ - 1},\,\,({\text{R}}^{2} = 0.998),$$

35 $${\text{FoM}} = 13.8439a^{2} + 26.9386a - 387.852,\,\,({\text{R}}^{2} = 0.9928),$$

36 $${\text{Q}} = - 4.45701a^{3} + 155a^{2} - 1231.85a + 3077.03,\,\,(R^{2} = 0.9994),$$

37 $${\text{SNR}} = - 0.332503a^{3} + 11.3595a^{2} - 89.991a + 223.06,\,\,({\text{R}}^{2} = 0.9994),$$

38 $${\text{DR}} = - 0.10417a^{3} + 2.99904a^{2} - 14.6576a + 46.2295,\,\,({\text{R}}^{2} = 0.9999),$$

39 $${\text{LoD}} = - 8.75462 \times 10^{ - 7} a^{3} + 3.20166 \times 10^{ - 5} a^{2} - 3.87932\times 10^{ - 4}a + 1.58471\times 10^{ - 3},\,\,({\text{R}}^{2} = 0.9928),$$

40 $${\text{RS}} = - 2.54804 \times 10^{ - 5} a^{3} + 9.1587\times 10^{ - 4}a^{2} - 1.08275\times 10^{ - 2}a + 4.24721\times 10^{ - 2},\,\,\,({\text{R}}^{2} = 0.9963).$$

So, the optimized value is chosen as 15 mm, where sensitivity, FoM, Q-factor, SNR, DR, LoD, and RS of 3.50 GHz/RIU, $3062$ /RIU, $4422$, 306, 150, $2\times {10}^{-5}$ RIU, 0.0002 are attained respectively.

### Effect of thickness of second MCP layer of the PhC (b):

**Figure 7:**
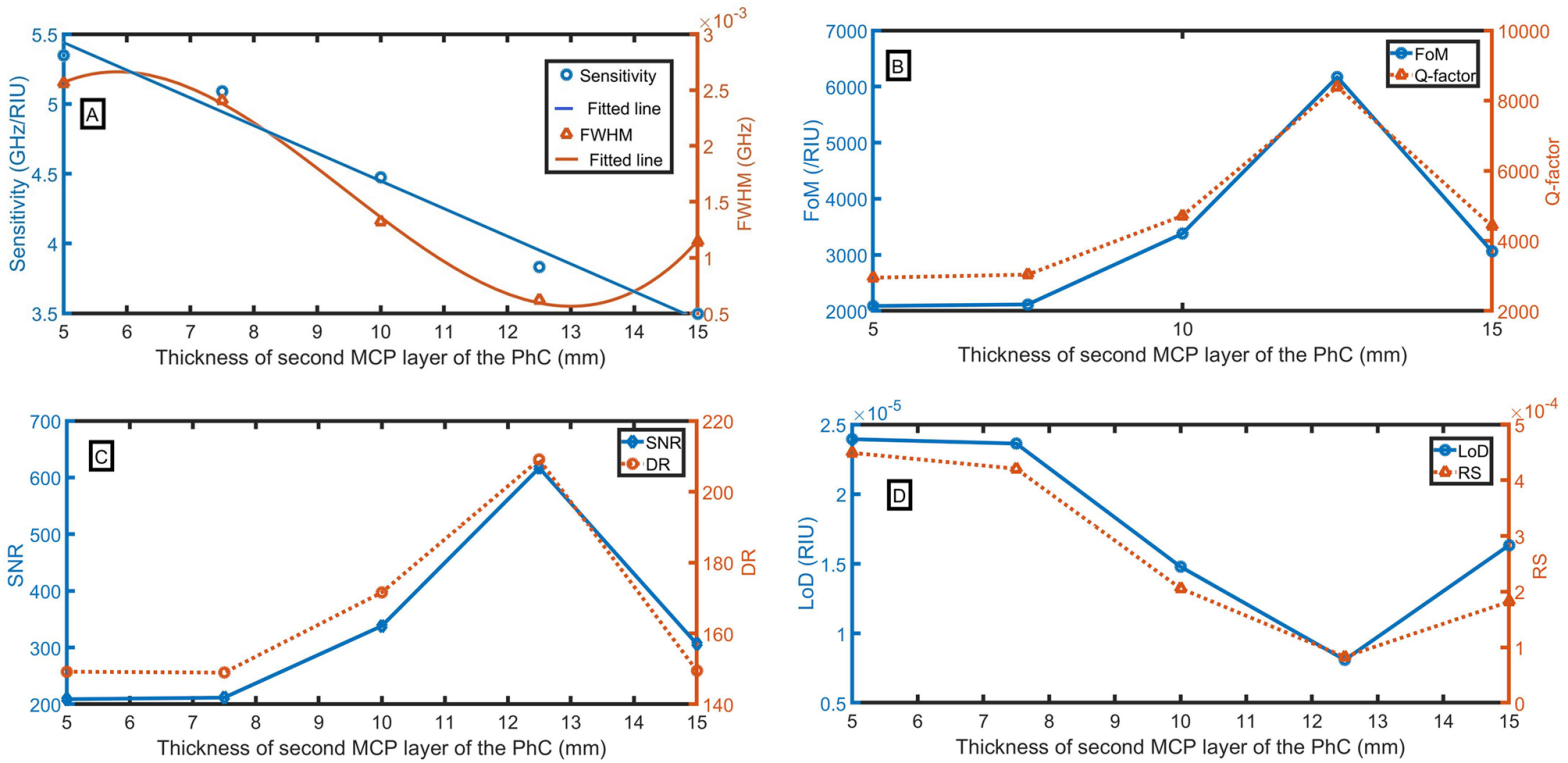
Evaluation of the sensor as a function of the thickness of the second MCP layer of the PhC with changing the sample refractive index from 1.0 to 1.1, (**A**) sensitivity and FWHM (for n = 1.1), (**B**) FoM and Q-factor, (**C**) SNR and DR, and (**D**) LoD and RS.

Additionally, the effect of thickness of even layers of MCPPhC (thickness of layer $a$) is scrutinized, which is represented in Fig. 7A–D. Here, it is perceived that the change in the thickness of layer $a$ has a significant impact on the sensing performance. The different performance parameters by changing the thickness of layer a are calculated from 5 to 15 mm and finally concluded that the sensitivity of the proposed sensor improves with the decrease of the thickness of a. By decreasing the thickness lower than 5 mm, the resonant peak goes out from the PBG. So, the optimized value of the thickness “a” is taken as 5 mm, where sensitivity, FoM, Q-factor, SNR, DR, LoD, and RS of 5.35 GHz/RIU, $2088$ /RIU, $2948$, 209, 149, $2\times {10}^{-5}$ RIU, 0.00045 are achieved, respectively. The following equations describe the relationship between the thickness of the second MCP layer of the PhC and S, FWHM:

41 $${\text{S}} = - 0.19828b + 6.4314,\,\,({\text{R}}^{2} = 0.9812),$$

42 $${\text{FWHM}} = 1.14745 \times 10^{ - 5} b^{ - 3} - 3.24445 \times 10^{ - 4}b^{2} + 2.61787\times 10^{ - 3}b - 3.84516\times 10^{ - 3},\,\,({\text{R}}^{2} = 0.9989).$$

### Effect of the incident angle:

**Figure 8:**
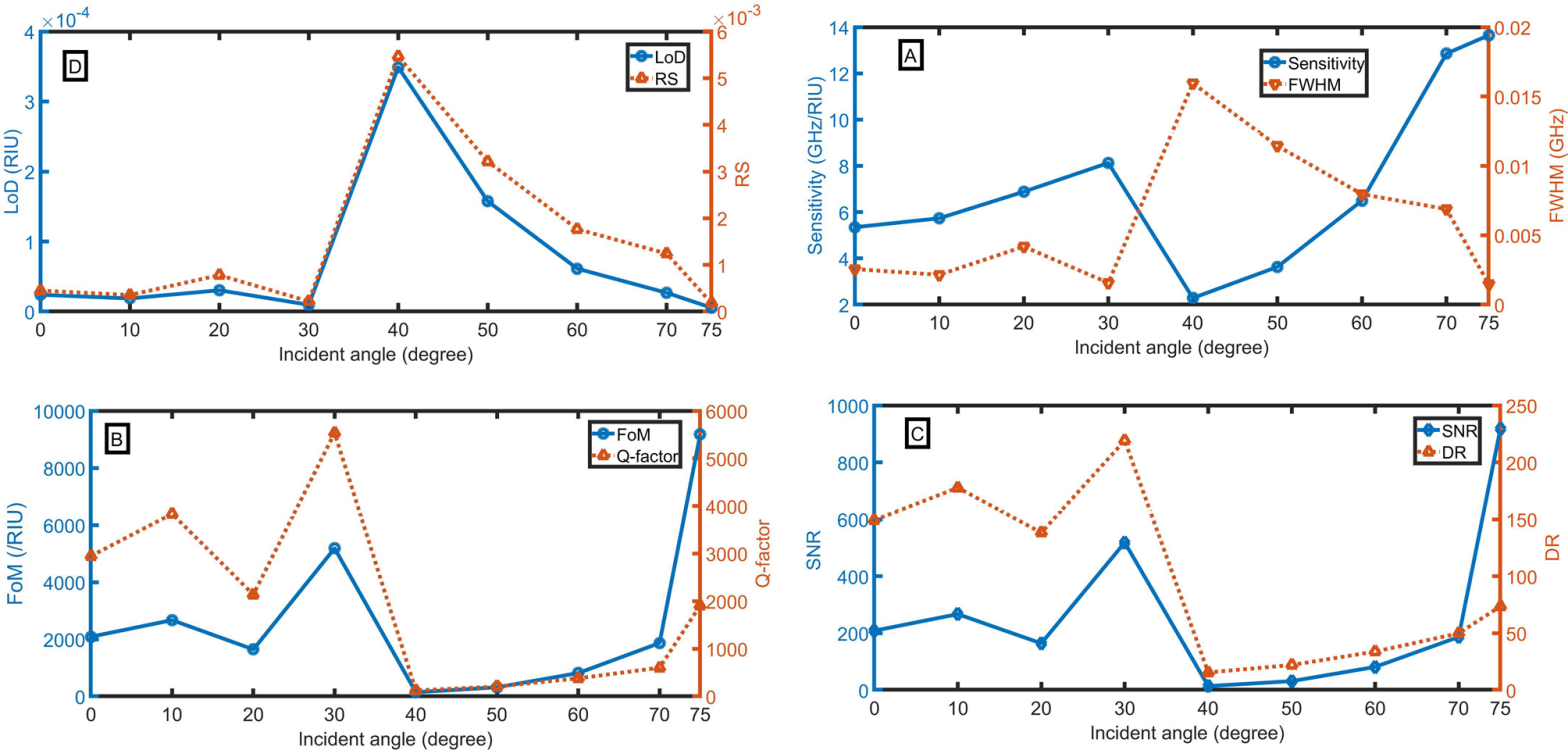
Evaluation of the sensor as a function of the incident angle with changing the sample refractive index from 1.0 to 1.1, (**A**) sensitivity and FWHM (for n = 1.1), (**B**) FoM and Q-factor, (**C**) SNR and DR, and (**D**) LoD and RS.

We also inspected the effect of incident angle variation from 0° to 75°, which is shown in Fig. 8A–D. During the analysis, we observed with increasing the incident angle from 0° to 30°, the resonant mode is shifted to high frequency. For the incident angle above 30°, multiple resonant modes are perceived. In addition to this, we noticed that above 75°, resonant modes overlap with each other. Further, we obtained the highest sensing performances at the incident angle of 75°. The optimum sensitivity, FoM, Q-factor, SNR, DR, LoD, and RS of 13.65 GHz/RIU, 9177 /RIU, $1909$, 918, 74, $5\times {10}^{-6}$ RIU, 2 $\times {10}^{-4}$ are accomplished, respectively. Therefore, we selected the angle 75° as the optimized incident angle.

**Figure 9:**
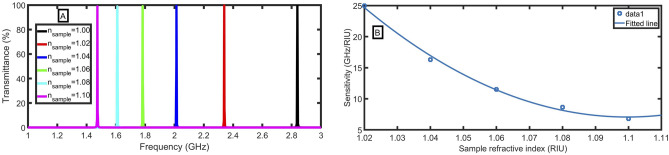
(**A**) The transmittance of the MCP-PhC structure as a function of frequency, and (**B**) the sensitivity with changing the sample refractive index from 1.0 to 1.1 at the optimum conditions.

By considering the optimized values of electron density, external magnetic field, the thickness of odd and even layers of the MCPPhC, thickness of the sample layer and incident angle, we investigated the transmittance characteristics of the proposed structure, as clear in Fig. 9A. The frequency of the defect mode is shifted to a lower frequency with increasing the refractive index of the sample analyte in the defect layer. Notably, the frequency is shifted from 2.84 to 1.47 GHz, as the sample refractive increases from 1.00 to 1.10. So, there is a remarkable frequency shift of 1.37 GHz for a 0.10 change in sample refractive index. Based on the above analysis, we computed sensitivity for each sample refractive index, which is plotted in Fig. 9B. The following equation describes the relationship between the sample refractive index and sensitivity:

43 $${\text{S}} = 2768.7n_{s}^{2} - 6089.6n_{s} + 3355.4,\,\,\;(R^{2} = 0.996).$$

A maximum sensitivity of 25 GHz/RIU is achieved with the optimized value of structure parameters at the refractive index from 1.00 to 1.02. In comparison to other studies, the suggested sensor has a high performance, as shown in Table 1.

**Table 1:** A comparison study between the proposed sensor and previous study (*NC* not counted).

References	S (GHz/RIU)	Q-factor	Materials
2014,^57^	2.998	814	positive and negative refractive index materials
2016,^58^	2	NC	Matrix of photonic molecules
2019,^59^	12.78	NC	Plasmonic hexagonal microstructured holes array in aluminum
2020,^60^	0.95	26.48	Enhanced toroidal localized spoof surface plasmons
2021,^61^	0.496	NC	Metamaterial for measuring electric permittivity
2021,^62^	0.78	162	Toroidal metasurface
This work	25	1909	1D-PC using MCP

## Conclusion

In summary, we have theoretically investigated the sensing perspective of 1D-MCPPhC. The proposed structure has a sandwiched defect layer between two similar types of PhCs. PhC is envisaged with an alternate layer of RHP and LHP magnetized cold plasma material. The transmittance spectrum is extensively scrutinized by manipulating the transfer matrix method. The mainstay of this research is to study the shifting nature of resonant mode with respect to change in analyte refractive index from 1.00 to 1.10. Numerous sensing performances like sensitivity, FoM, Q-factor, SNR, DR, LoD, and RS are meticulously analyzed under different values of electron density, external magnetic field, thickness of odd and even layer of the MCPPhC, thickness of the sample layer and incident angle. We obtained the optimized values as: ${n}_{e}=8\times {10}^{17} {\mathrm{m}}^{-3}$, B = 0.5 T, ${d}_{sample}=130 \mathrm{mm}$, ${d}_{a}={15 \mathrm{mm}, d}_{b}=5 \mathrm{mm}$, and incident angle = 75°. By considering these optimized values, an optimum sensitivity of 25 GHz/RIU can be achieved for the proposed sensor configuration.

## Data Availability

Requests for materials or code should be addressed to Z.A.Z.
